# Reduced GRAMD1C expression correlates to poor prognosis and immune infiltrates in kidney renal clear cell carcinoma

**DOI:** 10.7717/peerj.8205

**Published:** 2019-12-20

**Authors:** Haiyan Hao, Ziheng Wang, Shiqi Ren, Hanyu Shen, Hua Xian, Wenliang Ge, Wei Wang

**Affiliations:** 1 Department of Outpatient, Affiliated Hospital of Nantong University, Nantong, P.R. China; 2 Department of Medicine, Nantong University, Xinling college, Nantong, Jiangsu, P.R. China; 3 Department of Clinical Biobank, Nantong University Affiliated Hospital, Nantong, P.R. China; 4 Medical School of Nantong University, Nantong, P.R. China; 5 Department of Pediatric Surgery, Nantong University Affiliated Hospital, Nantong, Jiangsu, China

**Keywords:** Kidney renal clear cell carcinoma, Biomarkers, Bioinformatics, TCGA, Immune infiltrate, GRAMD1C

## Abstract

There has been an increase in the mortality rate and morbidity of kidney cancer (KC) with kidney renal clear cell carcinoma (KIRC) being the most common subtype of KC. GRAMD1C (GRAM Domain Containing 1C) has not been reported to relate to prognosis and immunotherapy in any cancers. Using bioinformatics methods, we judged the prognostic value of GRAMD1C expression in KIRC and investigated the underlying mechanisms of GRAMD1C affecting the overall survival of KIRC based on data downloaded from The Cancer Genome Atlas (TCGA). The outcome revealed that reduced GRAMD1C expression could be a promising predicting factor of poor prognosis in kidney renal clear cell carcinoma. Meanwhile, GRAMDIC expression was significantly correlated to several tumor-infiltrating immune cells (TIICs), particularly the regulatory T cells (Tregs). Furthermore, GRAMD1C was most significantly associated with the mTOR signaling pathway, RNA degradation, WNT signaling pathway, toll pathway and AKT pathway in KIRC. Thus, GRAMD1C has the potential to become a novel predictor to evaluate prognosis and immune infiltration for KIRC patients.

## Introduction

In all malignancies of adults worldwide, among which around 2% to 3% are afflicted by kidney cancer (KC) (Ferlay et al., 2010; Torre et al., 2015). Meanwhile, the highest incidence amongst all the urinary system tumors is the case of kidney cancer. There has been an increase in the mortality rate and morbidity of KC with kidney renal clear cell carcinoma (KIRC) being the most common subtype of KC (Cohen & McGovern, 2005). Due to the ineffective therapeutic methods available at present and late diagnosis, the survival rate of KIRC is far from ideal. Nevertheless, KIRC is known to be amongst the earliest malignancies that responded to immunotherapy and being most responsive (Escudier, 2012; Atkins, Regan & McDermott, 2004). The treatment of renal tumors over the last two decades has significantly improved with the emergence of the immune checkpoint blockade (ICB), indicating a positive direction in KIRC towards immunotherapy (Song et al., 2017). The prognosis and efficacy of immunotherapy and chemotherapy have been found to be influenced by tumor-infiltrating immune cells like tumor-infiltrating neutrophils and tumor associated macrophages (Zhang et al., 2018; Waniczek et al., 2017). However, there is very little data examining the relationship between the tumor characteristics and immune responses. Hence, for the development of fresh targeted therapies and efficient prognosis of KIRC, the identification of the immune-related biomarkers for examining the progression of the malignancy becomes imperative.

The GRAM domain is an intracellular protein-binding or lipid-binding signaling domain (Jiang, Ramamoorthy & Ramachandran, 2008). The importance of the GRAM domain was indicated by the mutations disrupting its phosphatase activity leading to X-linked myotubular myopathy in case of myotubularin (Doerks et al., 2000). Under tumorigenesis, the other members of the GRAM domain family were also shown to play a part (John et al., 2011). For instance, recently some reports implicated that GRAMD1B was responsible for chemo-resistance of the ovarian cancer patients, while the isolation of this gene resulted in anti-tumor effect in combination with paclitaxel synergistically (Wu et al., 2014). GRAMD1C (GRAM Domain Containing 1C), an uncharacterized protein belonging to the GRAM domain family of proteins, has not been reported to relate to prognosis and immune response in any cancers.  In present study, bioinformatic analysis using high throughput RNA-sequencing data from TCGA demonstrated that a reduced expression of GRAMD1C correlated to poor prognosis in KIRC.

Thus, the purpose of our present study was to evaluate the prognostic value of GRAMD1C expression in KIRC based on data downloaded from TCGA and investigated the underlying mechanisms of GRAMD1C affecting the prognosis of KIRC.

In our study, based on the gene expression profiles of KIRC and a comprehensive bioinformatics analysis, we analyzed the association of GRAMD1C with the characteristics of KIRC patients. Then, we investigated the correlation of GRAMD1C with TIICs in KIRC via a widely accepted evaluation algorithm, CIBERSORT. Moreover, we calculated the influence of different subtypes of TIICs on overall survival. The GSEA was performed finally with an intent to gain further breakthroughs in the underlying mechanism of GRAMD1C by identifying the biological pathways involved.

## Materials & Methods

### Data resources and data preprocessing

Gene expression profile and paired clinical information of KIRC patients, including 539 tumor samples and 72 para-carcinoma samples, were acquired from the Cancer Genome Atlas (TCGA) data portal (https://tcga-data.nci.nih.gov/tcga/), which serves as a public repository for archiving high-throughput microarray experimental data (Liu et al., 2018). The sequencing data of LUAD were generated with Illumina HiSeq_RNA-Seq platforms. In the subsequent processing, the trimmed mean of M values (TMM) normalization method was utilized for normalization of the downloaded data (Robinson, McCarthy & Smyth, 2010). The average expression data were calculated when met duplicate data. The processing process of the study fully satisfies the TCGA publication requirements. All preprocessing processes were realized by R software (version: 3.5.3) and Strawberry Perl. The processed expression data (Dataset S2) and script file for transformation and normalization of gene expression data (Dataset S3) have been uploaded into supplementary file. Cases lacking key clinical information, such as overall survival time, age, histologic grade (8 cases), gender, stage (3 cases), tumor status (T) (2 cases), and distant metastasis (M) (62 cases) were excluded. Then, we had to exclude all lymph node (N) data on account of the better part of KIRC cases downloaded from TCGA with no data on lymph node. Finally, mRNA expression level of 462 patients with kidney renal clear cell carcinoma and corresponding clinical information were reserved and used for further study. Table 1 uncovered KIRC patients’ characteristics were downloaded. Our study cohort, the mean age at diagnosis was 60 years old. Most patients were (*n* = 302, 65.4%) male, 160 (34.6%) were female. The histologic grade of KIRC in our study included undifferentiated (G4), poor-differentiated (G3), moderately-differentiated (G2) and well-differentiated (G1), separately taking up 1.9%, 42.0%, 41.1%, 14.9%. The tumor status contained 49.1% (*n* = 227) T1, 13.0% (*n* = 60) T2, 35.9% (*n* = 166) T3 and 1.9% (*n* = 9) T4. 73(15.8%) cases had distant metastasis and 389 (84.2%) had no-distant metastasis. At last contact, 307 (66.5%) subjects were alive, 155 (33.5%) were dead.

### CIBERSORT estimation

CIBERSORT is a widely used approach (Gentles et al., 2015; Ceccarelli et al., 2016) to characterize the cell composition of complex tissues based on their gene expression profiles, and it is highly consistent with ground truth estimations in many cancers (Newman et al., 2015). After uploading the gene expression data with standard annotation on to the CIBERSORT web portal (http://cibersort.stanford.edu/), the algorithm using the LM22 signature was run (Newman et al., 2015). LM22, the annotated gene signature matrix defining 22 immune cell subtypes, was downloaded from the CIBERSORT web portal (http://cibersort.stanford.edu/) (Ge et al., 2019). Then, CIBERSORT derived a *P*-value for the deconvolution for each sample using Monte Carlo sampling, providing a measure of confidence in the results (Newman et al., 2015). Instances where the CIBERSORT output was of *p*  < 0.05, it indicated that the inferred fractions of the immune cell populations produced by CIBERSORT were accurate (Anjum et al., 2016), and consequently further analysis with them was considered to be possible. For efficient comparison across the diverse samples, the CIBERSORT output were summarized to the Fig. S1, assisting in the visualization of the immune cell fraction of each sample. Types of immune cells could be sensitively and accurately discerned by CIBERSORT include T cells, B cells, macrophages, natural killer cells, dendritic cells and myeloid subsets. We grouped the samples into high and low GRAMD1C expressions based on median GRAMD1C expression value (1.922) to evaluate the difference of proportion of immune cells between high and low GRAMD1C expression.

**Table 1:** Kidney renal clear cell carcinoma patient characteristics in TCGA database.

Clinical characteristics		Total (462)	Percent
Age at diagnosis (years)		60(26–90)	
Gender	Female	160	34.6%
	Male	302	65.4%
Histologic grade	G1	9	1.9%
	G2	194	42.0%
	G3	190	41.1%
	G4	69	14.9%
Stage	Stage I	221	47.8%
	Stage II	49	10.6%
	Stage III	116	25.1%
	Stage IV	76	16.5%
Tumor status	T1	227	49.1%
	T2	60	13.0%
	T3	166	35.9%
	T4	9	1.9%
Distant metastasis	Negative (M0)	389	84.2%
	Positive (M1)	73	15.8%
Vital status	Dead	155	33.5%
	Alive	307	66.5%

### Identification of prognostic subtypes of TIICs in KIRC

We tried to identify the prognosis-related immune cell subtypes in KIRC. Based on the immune cell fraction of each sample evaluated by CIBERSORT analysis and clinical information acquired from TCGA database, we performed survival curves using “survival” package. Considering that clinical stage is a crucial factor determining prognosis of KIRCs, boxplots of clinical stage were performed using “ggplot2” package to visualize the association between the proportions of different types of TIICs and clinical stage.

### Gene set enrichment analysis

Gene set enrichment analysis (GSEA), a calculation method that could estimate whether a list of previous defined genes shows concordant differences with statistical significance between two biological processes (Subramanian et al., 2005). This study carried out the GSEA to elucidate the significant difference in survival rates observed between the low and high GRAMD1C groups after initially generating a sequential list of all genes according to their correlation to GRAMD1C expressions. For each analysis, the gene set permutations were performed 1000 times. The phenotype label was identified in the level of the GRAMD1C expression. In order to sort out the pathways enriched in each phenotype the Normalized Enrichment Score (NES), the nominal *p* value was utilized. The absolute value of NES>1.5 and *P* value < 0.05 were considered with statistical significance.

### Statistical analysis

All statistical analyses were performed by R (v.3.5.3). For evaluating the correlation between GRAMD1C expression and the other clinical characteristics (gender, age, histologic grade, clinical stage, tumor status and distant metastasis), we performed *χ*2 tests. The median GRAMD1C expression value determined the cut-off value of *χ*2 tests. The COX regression analysis was conducted to identify overall survival-related clinical characteristics in the TCGA patients. To study whether different proportions of tumor-infiltrating immune cells related to different clinical stages and diverse survival outcome, we performed boxplot and survival curve using “survival” and “ggplot2” packages (Wickham, 2011) based on results of CIBERSORT and clinical characteristics acquired from TCGA database. *P* value lower than 0.05 was considered statistically significant in this study.

## Results

### Correlation of the GRAMD1C expression with clinical characteristics

**Figure 1:**
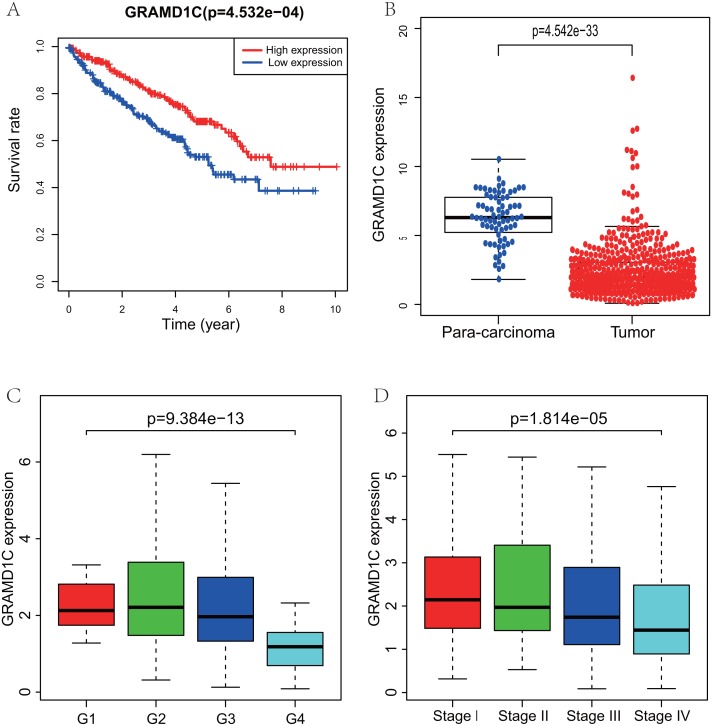
Correlations of GRAMD1C expression with several key clinicopathologic characteristics. (A) Decreased GRAMD1C expression significantly associates with poor overall survival. (B) The GRAMD1C expression in tumor tissues is obviously lower than in para-carcinoma tissues. (C) Decreased GRAMD1C expression significantly associates with unfavorable histologic grade. (D) Decreased GRAMD1C expression significantly associates with advanced clinical stage.

Using R (v.3.5.3), a total of 462 KIRC samples with GRAMD1C expression data and several patient characteristics (overall survival, histologic grade, clinical stage) were analyzed. Survival curve derived by “survival” package and boxplot of different histologic grade and clinical stages derived by “ggplot2” package were performed. Group cutoff value of survival curve was the median GRAMD1C expression (1.922). Figure 1 suggests that decreased GRAMD1C expression is significantly associated with poor overall survival (*P* < 0.01), advanced clinical stage (*P* < 0.01) and histologic grade (*P* < 0.01). In addition, the GRAMD1C expression in tumor tissues is obviously lower than that in para-carcinoma tissues (*P*-value < 0.01). In addition, *χ*2 tests (Table 2) reveal the relationship between the GRAMD1C expression and the clinical characteristics. The results demonstrate that the up-regulated of GRAMD1C expression in tumor tissues is significantly related to gender (*P* < 0.001), age (*P* = 0.041), histologic grade (*P* < 0.001), clinical stage (*P* = 0.001), tumor status (stage T, *P* = 0.001), distant metastasis (stage M, *P* = 0.007).

**Table 2:** Correlations between the expression of GRAMD1C and clinicopathological characteristics in KIRC.

Characteristic	*n*	Low or no expression	High expression	Pearson *χ*^2^	*P*
Total	462	231(50.00)	231(50.00)		
Gender				13.806	<0.001^*^
Male	302	170(56.30)	132(43.70)		
Female	160	61(38. 10)	99(61.90)		
Age				4.191	0.041^*^
≤60	230	104(45.20)	126(54.80)		
>60	232	127(54.70)	105(45.30)		
Histologic grade				33.168	<0.001^*^
I& II	203	84(41.40)	119(58.60)		
III	190	91(47.90)	99(52.10)		
IV	69	56(81.20)	13(18.80)		
Clinical stage				16.064	0.001^*^
I	221	91(41.20)	130(58.80)		
II	49	24(49.00)	25(51.00)		
III	116	67(57.80)	49(42.20)		
IV	76	49(64.50)	27(35.50)		
Stage T				13.098	0.001^*^
I	227	95(41.90)	13258.10)		
II	60	31(51.70)	29(48.30)		
III &IV	175	105(60.00)	70(40.00)		
Stage M				7.175	0.007^*^
0	389	184(47.30)	205(52.70)		
I	73	47(64.40)	26(35.60)		

**Notes.**

* *p* < 0.05.

### GRAMD1C is an independent predictor of prognosis in KIRC

**Figure 2:**
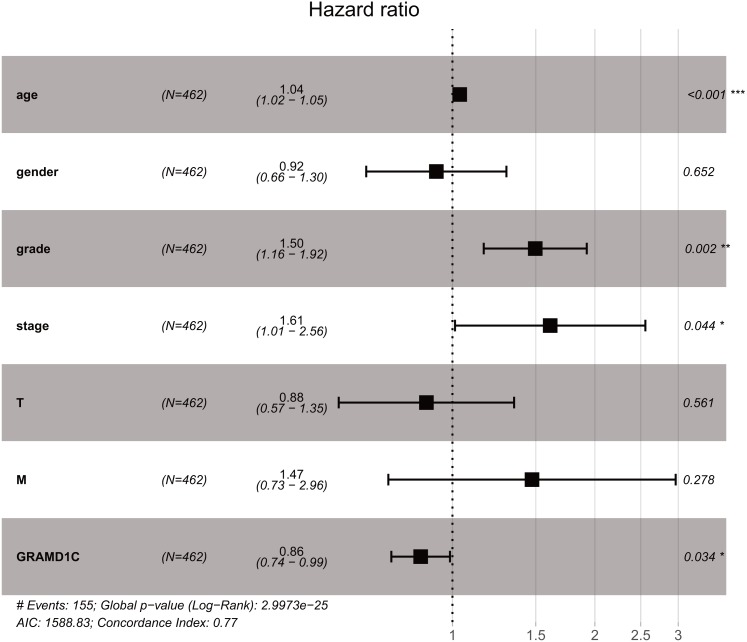
The outcome of Cox regression analysis. Multivariate analysis suggests that decreased GRAMD1C expression, advanced clinical stage and positive distant metastasis are individual predictors for poor prognosis in KIRC.

As shown in Table 3, Univariate analysis of correlation using Cox regression reveal that several factors, including age (HR = 1.03, *P*-value<0.01), histologic grade (HR = 2.38, *P*-value < 0.01), clinical stage (HR = 1.89, *P*-value < 0.01), tumor status (HR = 1.92, *P*-value < 0.01), distant metastasis (HR = 4.45, *P*-value < 0.01) as well as the GRAMD1C expression (HR = 0.72, *P*-value < 0.01) have significant correlation with overall survival. At multivariate analysis (Table 3B, Fig. 2), decreased GRAMD1C expression, advanced clinical stage, higher histologic grade and older age are individual predictors for poor prognosis.

**Table 3:** Results of Cox regression analysis. (A) Correlations of clinicopathologic characteristics with overall survival in KIRCs using Cox regression. (B) Multivariate survival model after variable selection.

Clinicopathologic variable	HR	HR.95L		HR.95H	*p* value
(A)					
Age	1.031	1.017		1.045	0.000
Gender	0.991	0.713		1.376	0.956
Grade	2.377	1.908		2.962	0.000
Stage	1.893	1.646		2.177	0.000
Tumor status (T)	1.920	1.611		2.287	0.000
Distant metastasis (M)	4.451	3.204		6.184	0.000
GRAMD1C	0.721	0.621		0.835	0.000
(B)					
Age	1.036	1.020		1.052	0.000
Grade	1.496	1.164		1.923	0.002
Stage	1.609	1.012		2.557	0.044
GRAMD1C	0.857	0.743		0.988	0.034

### GRAMD1C expression relates to the proportion of different types of TIICs in KIRC

**Figure 3:**
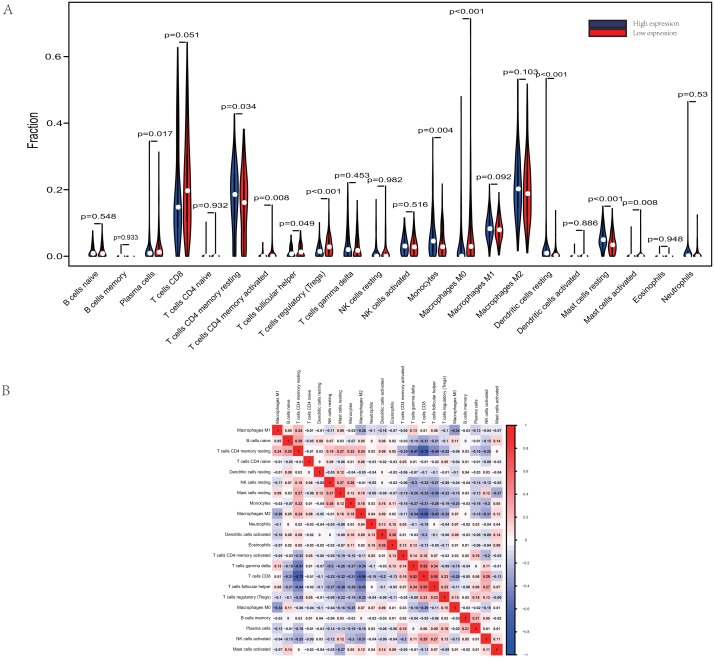
Evaluation of proportions of TIICs based on CIBERSORT. (A) The proportions of 22 tumor-infiltrating immune cells in high-GRAMD1C and low-GRAMD1C expression group. (B) Correlation matrix of all 22 immune cell proportions.

The proportions of TIICs vary substantially across immune subtypes and tumor types. Kidney renal clear cell carcinoma is one of tumors within the top leukocyte fraction and most responsive to immune checkpoint inhibitors (Thorsson et al., 2019). Therefore, we made an effort to find whether GRAMD1C expression has a correlation with immune infiltration level in KIRC. 462 tumor samples were split into 2 parts according to the median GRAMD1C expression (1.922) and a widely accepted computational resource (CIBERSORT) was used to infer the infiltrating level of 22 subtypes of immune cells. Barplot (Fig. S1) summarizes the outcome achieved from 462 KIRC patients. As shown in Fig. 3A and Table 4, plasma cells, T cells CD4 memory resting, T cells CD4 memory activated, T cells follicular helper, T cells regulatory (Tregs), monocytes, macrophages M0, dendritic cells resting, mast cells resting and mast cells activated significantly relate to the GRAMD1C expression. Among them, T cells CD4 memory resting (*p* = 0.034), monocytes (*p* = 0.004), dendritic cells resting (*p* < 0.001) and mast cells resting (*p* < 0.001) are apparently increased in high expression group relative to low expression group. In contrast, the proportions of plasma cells (*p* = 0.017), T cells follicular helper (*p* = 0.049), T cells regulatory (Tregs) (*p* < 0.001), macrophages M0 (*p* < 0.001) are lower in high expression group. The outcome intensely suggests that GRAMD1C plays a role in regulating the formation of immune microenviroment in kidney renal clear cell carcinoma, especially impacting on those subtypes of T cells and macrophages. Proportions of different types of immune cells subsets were weakly and then moderately correlated (Fig. 3B). T cells follicular helper and T cells CD8 displayed the strongest positive correlation (Pearson correlation = 0.55), while T cells CD8 and T cells CD4 memory resting showed the strongest negative correlation (Pearson correlation = 0.55).

**Table 4:** Relative proportions of 22 types of immune cells in high and low risk score groups.

Immune cell type	Low GRAMD1C group	High GRAMD1C group	*P* values
B cells naive	1.53% ± 1.73%	1.55% ± 1.88%	0.548
B cells memory	0.04% ± 0.29%	0.01% ± 0.06%	0.933
Plasma cells	1.91% ± 3.57%	2.96% ± 4.67%	0.017
T cells CD8	19.76% ± 14.89%	21.86% ± 14.38%	0.051
T cells CD4 naive	0.04% ± 0.58%	0.03% ± 0.48%	0.82
T cells CD4 memory resting	17.74% ± 9.67%	15.69% ± 9.77%	0.034
T cells CD4 memory activated	0.40% ± 0.89%	0.82% ± 1.96%	0.008
T cells follicular helper	1.33% ± 1.58%	1.71% ± 1.86%	0.049
T cells regulatory (Tregs)	1.86% ± 1.83%	3.30% ± 2.83%	<0.001
T cells gamma delta	3.78% ± 4.76%	3.07% ± 3.64%	0.453
NK cells resting	1.22% ± 2.83%	1.37% ± 3.36%	0.982
NK cells activated	3.40% ± 2.67%	3.30% ± 2.79%	0.516
Monocytes	5.90% ± 5.90%	4.28% ± 4.25%	0.004
Macrophages M0	3.02% ± 6.24%	6.32% ± 9.29%	<0.001
Macrophages M1	8.64% ± 3.78%	8.00% ± 3.45%	0.092
Macrophages M2	21.16% ± 9.45%	19.78% ± 9.41%	0.103
Dendritic cells resting	1.83% ± 4.22%	0.82% ± 1.57%	<0.001
Dendritic cells activated	0.13% ± 0.52%	0.18% ± 0.85%	0.886
Mast cells resting	5.45% ± 3.14%	3.92% ± 2.96%	<0.001
Mast cells activated	0.12% ± 0.81%	0.31% ± 1.42%	0.0089
Eosinophils	0.06% ± 0.30%	0.04% ± 0.24%	0.948
Neutrophils	0.72% ± 3.37%	0.70% ± 1.54%	0.530

### Identification of prognostic subtypes of TIICs in KIRC

**Figure 4:**
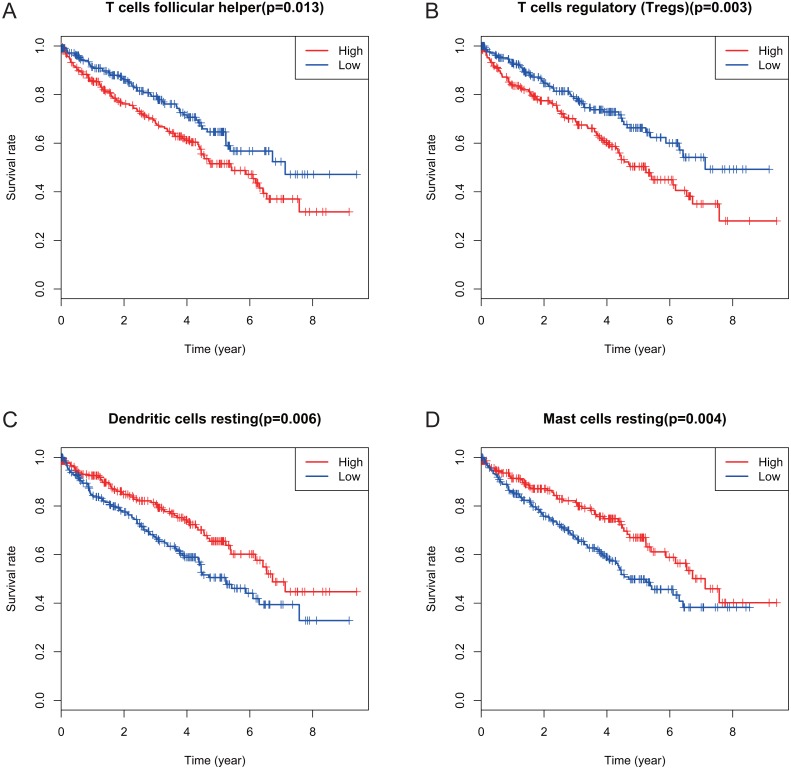
Correlations between the proportions of TIICs and overall survival. (A) Decreased proportion of T cells follicular helper is significantly associated with higher overall survival of KIRC (*p* = 0.013). (B) Decreased proportion of T cells regulatory (Tregs) is significantly associated with higher overall survival of KIRC (*P* = 0.003). (C) Increased proportion of dendritic cells resting is significantly associated with higher overall survival of KIRC (*p* = 0.006) (D) Increased proportion of Mast cells resting is significantly associated with higher overall survival of KIRC (*p* = 0.004).

**Figure 5:**
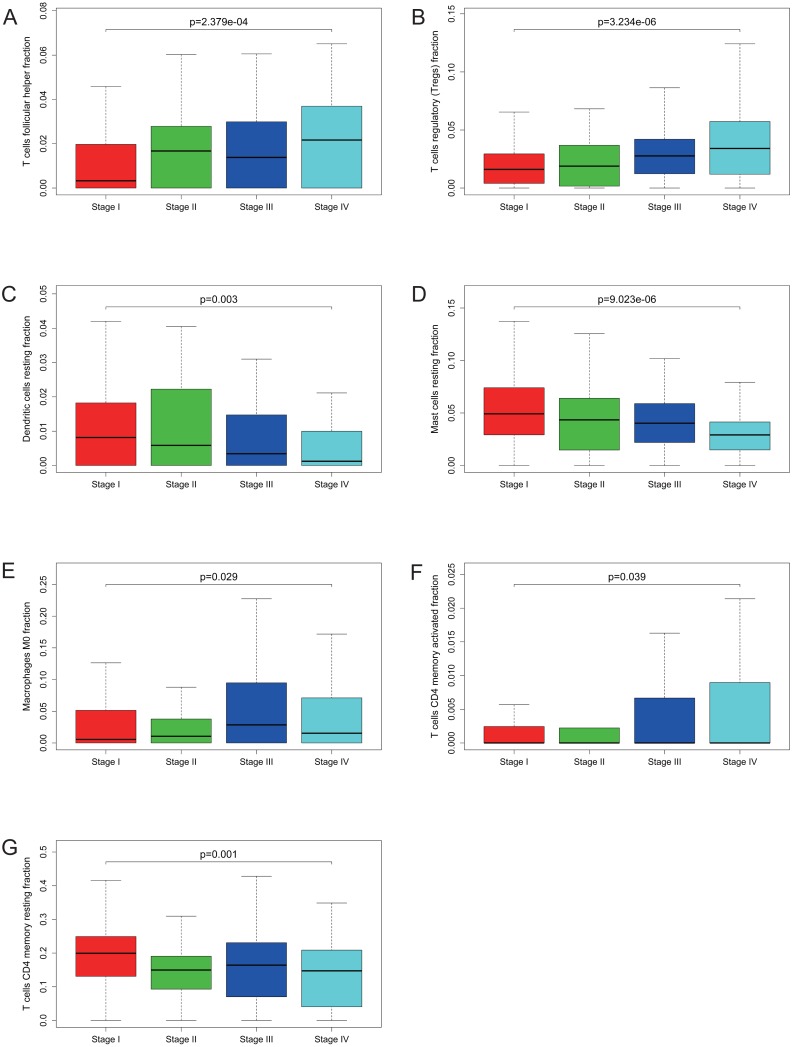
Correlations between the proportions of TIICs and clinical stage. T cells follicular helper (A), T cells regulatory (Tregs) (B), dendritic cells resting (C), mast cells resting (D), macrophages M0 (E), T cells CD4 memory activated (F) and T cells CD4 memory resting (G) are significantly correlated with clincial stage in KIRC.

Many previous studies have demonstrated that the proportions of TIICs are reliable predictors of prognosis (Dunn, Dunn & Curry, 2007; Clemente et al., 1996). Thus, we tried to identify the prognostic subtypes of TIICs in KIRC. Based on lymphocyte fraction of each sample evaluated by CIBERSORT and clinical information (overall survival and clinical stage) acquired from TCGA, using R (v.3.5.3), we graphed survival curve and clinical staging boxplot of 22 types of different immune cells. Detailed outcome was shown in Figs. S2 and S3. Selected survival curves and clinical staging boxplots of subtypes of immune cells which relate to GRAMD1C expression (*p* < 0.05) were exhibited in Figs. 4 and 5. The outcome uncovered that T cells follicular helper (*p* = 0.013), T cells regulatory (Tregs) (*p* = 0.003), dendritic cells resting (*p* = 0.006) and mast cells resting (*p* = 0) were 4 types of immune cells which were associated with overall survival of KIRC patients. Meanwhile, according to the results of CIBERSORT, these 4 types of immune cells were all related to GRAMD1C expression. B cells memory (*p* = 0.048), T cells CD8 (*p* = 0.004), T cells CD4 memory resting (*p* = 0.001), T cells CD4 memory activated (*p* = 0.039), T cells follicular helper (*p* = 0.000), T cells regulatory (Tregs) (*p* = 0.000), T cells gamma delta (*p* = 0.032), NK cells resting (*p* = 0.007), macrophages M0 (*p* = 0.029), macrophages M2 (*p* = 0.000), dendritic cells resting (*p* = 0.003) and mast cells resting (*p* = 0.000) were correlated with clinical stage of KIRC, thereinto T cells CD4 memory resting, T cells CD4 memory activated, T cells follicular helper, T cells regulatory (Tregs), macrophages M0, mast cells resting and dendritic cells resting are GRAMD1C-related immune cells. In general, T cells follicular helper and T cells regulatory (Tregs) tend to be associated with poor outcome and advanced clinical stage, consistent with proposed role of Tregs as pro-tumourigenic immune suppressors (Shimizu, Yamazaki & Sakaguchi, 1999; Onizuka et al., 1999). In contrast, mast cells resting and dendritic cells resting relate to favourable prognosis and clinical stage.

### Gene set enrichment analysis

**Figure 6:**
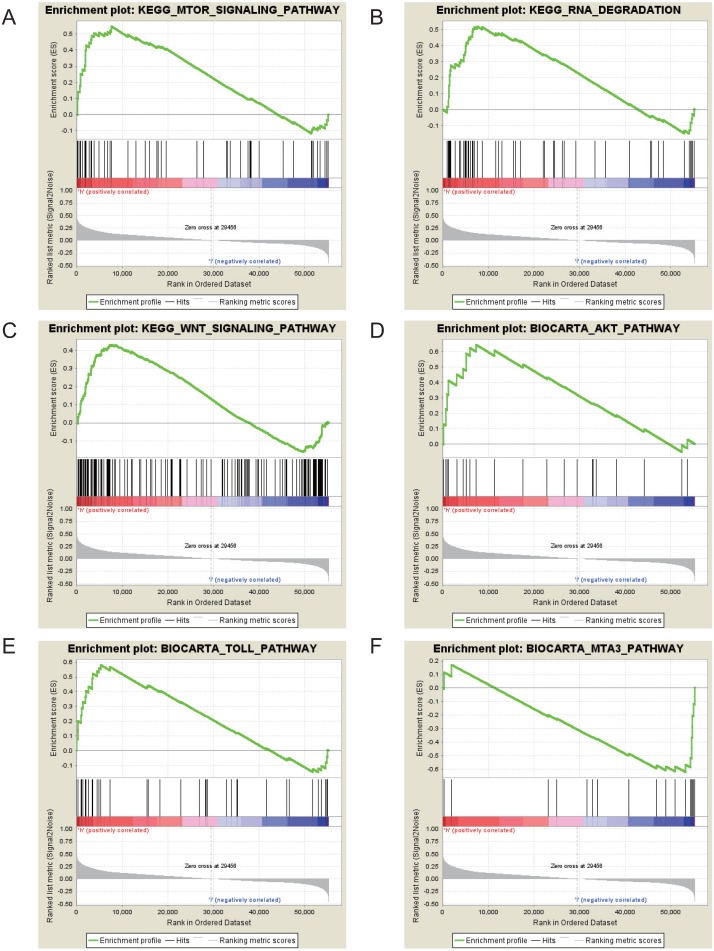
Enrichment plots from gene set enrichment analysis (GSEA). mTOR signaling pathway (A), RNA degradation (B), WNT signaling pathway (C), AKT pathway (D), Toll pathway (E) and MTA3 pathway (F) are differentially enriched in GRAMD1C-related KIRC.

The Gene Set Enrichment Analysis (GSEA) between high and low GRAMD1C expression data sets was conducted to identify the signaling pathways differentially activated in KIRC. In the enrichment analysis of MSigDB Collection (c2.cp.biocarta and c2.cp.kegg), significant differences caused by GRAMD1C were revealed in the GSEA (*p*-value  < 0.05). According to their normalized enrichment scores (NES) we selected the most highly enriched signaling pathways (Fig. 6 and Table 5). The outcome revealed that mTOR signaling pathway, RNA degradation, WNT signaling pathway, toll pathway and AKT pathway were differentially enriched in high GRAMD1C expression phenotype. MTA3 pathway was enriched in low expression phenotype.

## Discussion

Renal clear cell carcinoma (KIRC) is the most common kidney renal cell cancer (Srigley et al., 2013). Despite advances in diagnosis, screening, surgery and drug therapy, the clinical outcome of KIRC remains unsatisfactory. Immune response is closely associated with clinical outcome in KIRC. Tumor- infiltrating immune cells (TIICs) form an ecosystem in the tumor microenvironment to regulate cancer progression and have shown potential prognostic value (Grivennikov, Greten & Karin, 2010). GRAMD1C is a messenger RNA which has not been reported to relate to prognosis and respond to immunotherapy in any cancers. Here, based on mining the gene expression profiles of KIRC and a comprehensive bioinformatics analysis, we found that the expression of GRAMD1C is correlated to leukocyte fraction, especially the regulatory T cell (Tregs) and overall survival. In addition, it is potential to serve as a predictor of prognosis for KIRC patients.

**Table 5:** Gene sets enrichment.

MSigDB collection	Gene set name	NES	*p*-val
c2.cp.kegg.v6.2.symbols.gmt	KEGG_MTOR_SIGNALING_PATHWAY	1.987	0.004
c2.cp.biocarta.v6.2.symbols.gmt	BIOCARTA_AKT_PATHWAY	1.911	0.006
	KEGG_RNA_DEGRADATION	1.823	0.025
	BIOCARTA_TOLL_PATHWAY	1.774	0.033
	KEGG_WNT_SIGNALING_PATHWAY	1.679	0.041
	BIOCARTA_MTA3_PATHWAY	−1.846	0.004

**Notes.**

NES normalized enrichment score

Gene sets with p-val smaller than 0.05 were considered.

The reduced expression of GRAMD1C in KIRC was established to be associated with advanced clinical pathologic characteristics (high clinical stage, distant metastasis, and bad histological grade), poor prognosis and lower overall survival through the bioinformatic analysis with high throughput RNA-sequencing data from TCGA by this study. The multivariate analysis suggested GRAMD1C is an independent predictor of overall survival. To further investigate the functions of GRAMD1C in KIRC, we performed immune-related analysis using CIBERSORT, which showed that the GRAMD1C expression significantly effects the leukocyte fraction in tumor microenvirenment of KIRC. In addition, based on clinical information acquired from TCGA and CIBERSORT results, we found that several tumor-infiltrating immune cells related to GRAMD1C expression have significant correlation with prognosis of KIRC patients, which is consistent with previous studies (Xu et al., 2013; Wang & Ke, 2011; Overacre-Delgoffe et al., 2017). More interestingly, the GRAMD1C expression has a negative correlation with the regulatory T cell (Tregs) and T cells follicular helper which predicted a poor clinical outcome and advanced clinical stage. Another important aspect of this study was that GRAMD1C expression was correlated with diverse well-known pathways associated with cancer processes and immune responses, such as mTOR signaling pathway, WNT signaling pathway and AKT pathway.

Several previous studies could explain why GRAMD1C expression correlates to the poor prognosis and the proportion of regulatory T cells (Tregs). The activation of the tumor-infiltrating immune cells was affected by the mTOR/AKT pathway as examined by the research in the field of immunology in the last decade. The Akt-mTOR axis has been widely recognized as the critical negative regulator of the regulatory T cell (Tregs) de novo differentiation in the regulatory T cell (Tergs) compartment (Delgoffe et al., 2009; Sokol et al., 2008; Sauer et al., 2008; Liu et al., 2010) with growth in population (Battaglia, Stabilini & Roncarolo, 2005). Interestingly, our present study demonstrated that over expression of GRAMD1C in KIRC was associated with activated Akt/mTOR pathway and reduced proportion of the regulatory T cell (Tregs). Thus, in the writer’s view, GRAMD1C regulates the regulatory T cells by activating mTOR/AKT pathway, but the exact molecular mechanism needs to be further studied. This may partially explain why reduced expression of GRAMD1C relates to poor prognosis in KIRCs. Another important pathway we have discovered associated with GRAMD1C is MTA3 pathway. Researchers have manifested that MTA3 can serve as a regulatory factor of the proteins of p-PARP, BAX, Cleved-Caspase-3 and Bcl-2 to accelerate the cellular apoptosis in NSCLCs (Li et al., 2015). It has also been described as an independent biomarker for unfavorable prognosis in hepatocellular carcinoma (Wang et al., 2017) and uterine non-endometrioid carcinomas (Mylonas & Bruning, 2012). In present study, the results of GSEA established a correlation of low GRAMD1C expression phenotype and intense MTA3 signaling, which was associated with poor prognosis.

The most interesting thing we found about this study is the regulation of GRAMD1C on tumor-infiltrating immune cells, especially the regulatory T cells (Tregs). The Terg cells are implicated in a range of medical conditions like cancer and other autoimmune diseases, but are also known to be the immunosuppressive subset of CD4+ T cells that maintain the immune homeostasis by regulating the numerous facets of immune response (Sakaguchi et al., 1995; Khattri et al., 2003). The various types of effector lymphocytes are suppressed by the Treg cells migrating into the inflammatory sites (Ashutosh et al., 2009; Yeonseok et al., 2011; Koch et al., 2009; Linterman et al., 2011). Treg cells are often found in inflamed tumors harboring large numbers of TH cells and CTLs with regard to cancer (Williams et al., 2017). In tumorigenesis the development of the immune escape mechanisms is a significant factor involving the recruitment and/or induction of the Tergs, programmed cell death 1 ligand 1 (PD-L1), programmed cell death 1 (PD-1), and the immunosuppressive cells (Schreiber, Old & Smyth, 2011). Hence, by depleting or inhibiting these immunosuppressive factors the responses of the anti-tumor immune system could be potentially unleashed. The reinvigorate dysfunctional or ‘exhausted’ cytotoxic CD8+ T cells can be enabled to attack the cancer cells and counter the immunosuppression by anticancer immunotherapy with immune checkpoint inhibitors (ICIs) (Zou, Wolchok & Chen, 2016; Pardoll, 2012); anti-PD-L1 monoclonal antibodies (mAbs), anti-PD-1 and anti-CTLA-4, have shown remarkable clinical efficacy across a range of different cancers, with advanced-stage disease patients (Topalian et al., 2012) (Taneja, 2012). More effective therapies along with immunotherapy combinations, are urgently needed as in majority of the cases the efficacy of the ICIs have been found to be unsatisfactory. Considering the fact that by suppressing the antitumor immunity the Treg cells can promote tumor progression (Shimon et al., 2010; Wing & Sakaguchi, 2010), by targeting the immunosuppressive factors or by manipulating Tregs can be looked upon as a new anticancer treatment methodology with promising results (Onizuka et al., 1999; Shimizu, Yamazaki & Sakaguchi, 1999). Thus, Considering the regulatory effect of GRAMD1C on the infiltration of Tregs, GRAMD1C is likely to play a role in promoting immunologic escape in KIRC. However, the exact biologic process needs to be further demonstrated.

Our study, for the first time, established a correlation of reduced GRAMD1C with poor prognosis of KIRC and higher infiltration level of several types of immune infiltrating immune cells and discovered that GRAMD1C was likely could serve as a new prognostic marker for KIRC patients. Nonetheless, the prediction of protein expression using mRNA was found to be very unreliable as formulated by Guo et al. (2008). The correlation between GRAMD1C protein and the GRAMD1C mRNA expression could not be assessed clearly due to the limitations in the design of this study. The concept of GRAMD1C needs to be explored further.

## Conclusions

Our study indicated that reduced GRAMD1C expression correlates with diverse clinical characteristics (gender, age, histologic grade, clinical stage, tumor status and distant metastasis). Reduced GRAMD1C expression is an independent predicting factor of poor prognosis in kidney renal clear cell carcinoma. Meanwhile, GRAMDIC expression significantly correlates with several tumor-infiltrating immune cells, particularly the regulatory T cells (Tregs). Moreover, the mTOR signaling pathway, RNA degradation, WNT signaling pathway, toll pathway and AKT pathway serve as the major pathway affected by GRAMD1C in KIRC. Above all, GRAMD1C is a promising biomarker of prognosis and correlates with immune infiltration in KIRC.

##  Supplemental Information

10.7717/peerj.8205/supp-1 Figure S1 The immune cell fraction of each samples The barplot summarizes the outcome achieved from CIBERSORT analysis of 462 KIRC patients. The lengths of columns represnt the relative proportions of immune cells. The colors of columns represent the subtypes of immune cells.

10.7717/peerj.8205/supp-2 Figure S2 Correlations between the proportions of 22 subtypes of immune cells and overall survival Each survival curve reveals the correlation between the relative proportion of this subtype of immune cells and overall survival in KIRC.

10.7717/peerj.8205/supp-3 Figure S3 Correlations between the proportions of 22 subtypes of immune cells and clinical stage Each histogram reveals the correlation bewteen the relative proportion of immune cellsand clinical stage.

10.7717/peerj.8205/supp-4 Dataset S1 The processed clinical information The processed clinical information acquired from TCGA. Cases lacking key clinical information, such as overall survival time, age, histologic grade (8 cases), gender, clinical stage (3 cases), tumor status (T) (2 cases), and distant metastasis (M) (62 cases) were excluded.

10.7717/peerj.8205/supp-5 Dataset S2 The processed gene expression profile of kidney renal clear cell carcinoma The processed gene expression profile of kidney renal clear cell carcinoma tissues and para-carcinoma tissues. The row data was acquired from TCGA database.

10.7717/peerj.8205/supp-6 Supplemental Information 1 The scripts of R software and Strawberry Perl for transformation and normalization of gene expression data The scripts of R software and Strawberry Perl for transformation and normalization of gene expression data.

## Additional Information and Declarations

### Data Availability

The following information was supplied regarding data availability: The raw data was downloaded from the publicly available TCGA database: search term TCGA-KIRC.
