# Supplementary material for: Reduced GRAMD1C expression correlates to poor prognosis and immune infiltrates in kidney renal clear cell carcinoma
Source: PeerJ. 2019 Dec 20;7:e8205. doi: 10.7717/peerj.8205 (PMC6927341; doi:10.7717/peerj.8205)
Supplement: Figure S1 — The barplot summarizes the outcome achieved from CIBERSORT analysis of 462 KIRC patients. The lengths of columns represnt the relative proportions of immune cells. The colors of columns represent the subtypes of immune cells. [file peerj-07-8205-s001.pdf]

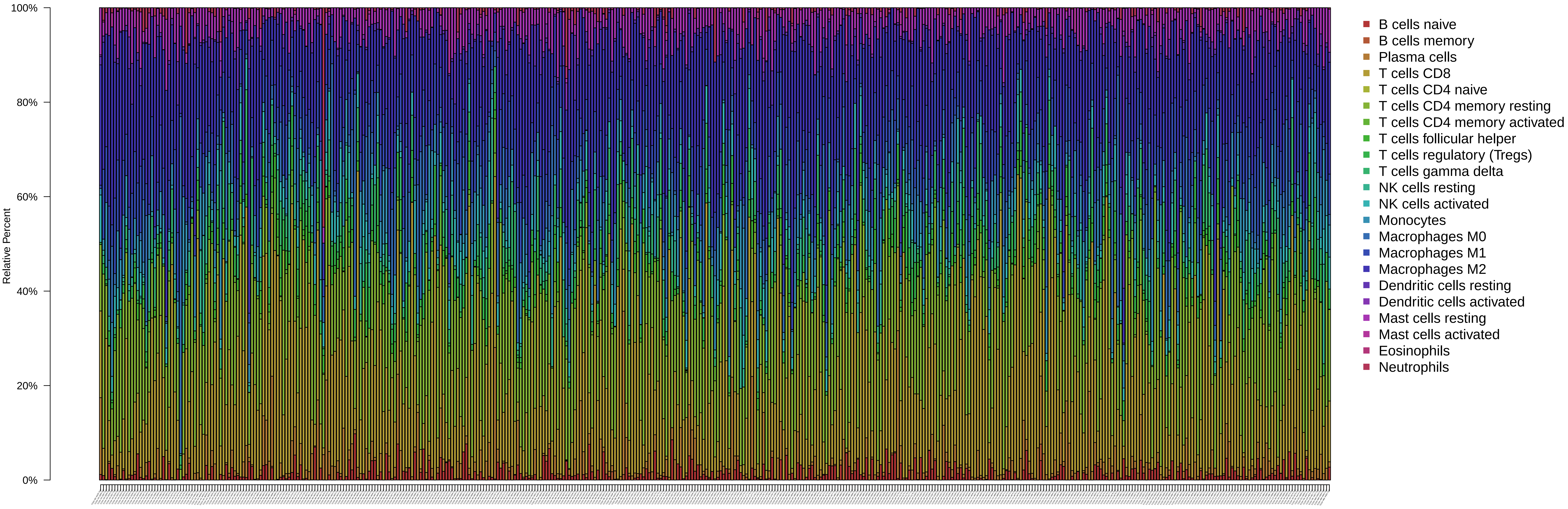

Relative Percent
100%
80%
60%
40%
20%
0%
B cells naive
B cells memory
Plasma cells
T cells CD8
T cells CD4 naive
T cells CD4 memory resting
T cells CD4 memory activated
T cells follicular helper
T cells regulatory (Tregs)
T cells gamma delta
NK cells resting
NK cells activated
Monocytes
Macrophages M0
Macrophages M1
Macrophages M2
Dendritic cells resting
Dendritic cells activated
Mast cells resting
Mast cells activated
Eosinophils
Neutrophils
