# Supplementary figures and images for: Reduced GRAMD1C expression correlates to poor prognosis and immune infiltrates in kidney renal clear cell carcinoma
Source: PeerJ. 2019 Dec 20;7:e8205. doi: 10.7717/peerj.8205 (PMC6927341; doi:10.7717/peerj.8205)

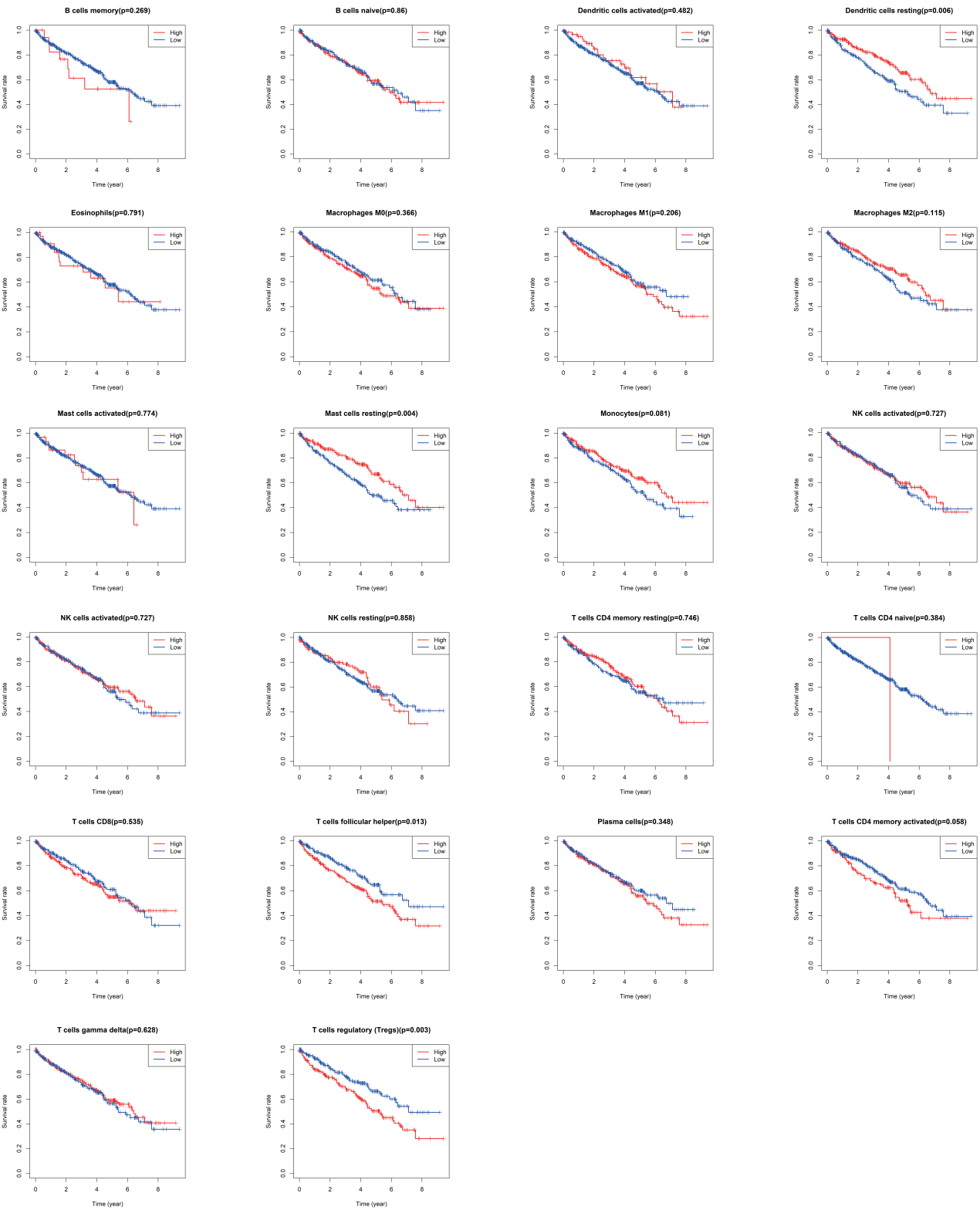

Supplement: Figure S2 — Each survival curve reveals the correlation between the relative proportion of this subtype of immune cells and overall survival in KIRC. [file peerj-07-8205-s002.pdf]

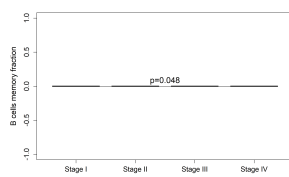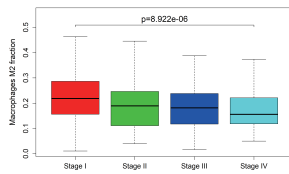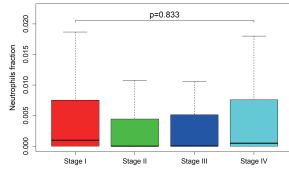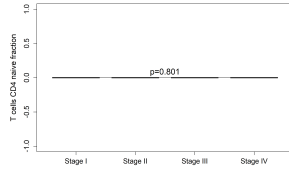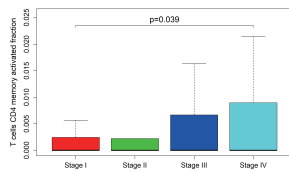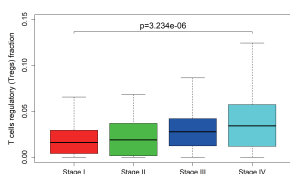

Supplement: Figure S3 — Each histogram reveals the correlation bewteen the relative proportion of immune cellsand clinical stage. [file peerj-07-8205-s003.pdf]
